# Energy-Efficient and Fast Data Gathering Protocols for Indoor Wireless Sensor Networks

**DOI:** 10.3390/s100908054

**Published:** 2010-08-27

**Authors:** Abdullah Erdal Tümer, Mesut Gündüz

**Affiliations:** 1 Department of Computer Education & Instructional Technology, Faculty of Education, University of Selcuk, 42090 Meram, Konya, Turkey; E-Mail: tumer@selcuk.edu.tr; 2 Department of Computer Engineering, Faculty of Engineering, University of Selcuk, 42075 Kampus, Konya, Turkey; E-Mail: mgunduz@selcuk.edu.tr

**Keywords:** wireless sensor networks, routing protocols, data gathering, reactive applications, event-driven, critical threshold, xor, data aggregation and filtering

## Abstract

Wireless Sensor Networks have become an important technology with numerous potential applications for the interaction of computers and the physical environment in civilian and military areas. In the routing protocols that are specifically designed for the applications used by sensor networks, the limited available power of the sensor nodes has been taken into consideration in order to extend the lifetime of the networks. In this paper, two protocols based on LEACH and called R-EERP and S-EERP with base and threshold values are presented. R-EERP and S-EERP are two efficient energy aware routing protocols that can be used for some critical applications such as detecting dangerous gases (methane, ammonium, carbon monoxide, *etc*.) in an indoor environment. In R-EERP, sensor nodes are deployed randomly in a field similar to LEACH. In S-EERP, nodes are deployed sequentially in the rooms of the flats of a multi-story building. In both protocols, nodes forming clusters do not change during a cluster change time, only the cluster heads change. Furthermore, an XOR operation is performed on the collected data in order to prevent the sending of the same data sensed by the nodes close to each other. Simulation results show that our proposed protocols are more energy-efficient than the conventional LEACH protocol.

## 1. Introduction

A wireless sensor network is composed of a large number of tiny sensor nodes [1]. The main task of a wireless sensor node is to sense and collect data from a certain region, process them and transmit them to a sink node where further processing on the collected data can be performed [2]. Sensor nodes are small-scale and cost effective devices with limited capabilities. Wireless sensor networks generally contain thousands of sensor nodes, which are randomly deployed to a field. The sensor nodes are powered by batteries and controlled remotely. For most applications, it is impossible to recharge or replace the batteries of sensor nodes after deployment [3].

Depending on the application of sensor networks, certain routing protocols are required in order to establish the communication among sensor nodes and the sink nodes [4]. Wireless sensor networks consume their limited energy for collecting data, performing calculations and routing the received data. Nevertheless, in most applications, each sensor node is expected to last for a long time [5]. For this reason, both efficient use of energy and efficient routing schemes are highly important in sensor networks [3].

Various routing protocols have been proposed for conventional wireless *ad hoc* networks. But these protocols are not fully suited to the unique features and application requirements of sensor networks [6]. Hence, new routing algorithms for sensor networks are also proposed in the literature.

Routing algorithms for *ad-hoc* networks are typically classified as either proactive or reactive [7]. Similarly, routing protocols for sensor networks can be categorized as proactive and reactive as well. Proactive routing protocols will have routing paths established/determined from all nodes to sink all the time. Hence they are very suitable for continuous monitoring applications where all parts of a region are to be monitored all the time. In such applications, all sensor nodes sense and send data to the sink node periodically. LEACH (Low Energy Adaptive Clustering Hierarchy) [8] is a good approximation of a proactive network protocol.

Reactive routing protocols do not have to establish paths from all nodes to the sink all the time. They may establish paths when required. For example, the Directed Diffusion [9] routing protocol establishes paths from some sensor nodes to the sink upon sending of queries from sink to nodes. This type of routing protocols can be used for event-driven or query-driven sensor network applications. These applications can also be called as reactive applications. In such applications, data is sent from one or more sensor nodes when there are certain events happened or when queries are sent. Not all nodes have data to send to the sink at each period of time. Event though the communication and routing can be reactive, in such applications nodes may need to sense their environment continuously to detect suddenly happening events. For example, the TEEN (Threshold-sensitive Energy Efficient sensor Network) protocol [10] has been developed specifically for such networks. While proactive algorithms are convenient for applications that require periodical observations, reactive algorithms are convenient for applications where sudden changes are considered to be important.

In this paper we follow a hybrid approach and propose two routing protocols that are a combination of the proactive and reactive approaches. Our protocols are designed for reactive emergency applications. Hence, we do not transport data to the sink node all the time. We transfer with some certain rules, so in this sense it is reactive. However, since we are dealing with emergency applications, when there is a need, the data has to be transported to the sink node as soon as possible. For this reason we should have the routing paths to be established earlier, proactively. Therefore, our approach has both features. It is proactively building the routing paths, but using them reactively. Hence it is a solution that is good for not only periodic data gathering, but also for reactive data gathering. The proactive building of the paths is similar to the LEACH, it is cluster based, and uses a two-level hierarchy. Hence, all the paths from nodes to the sink are just 2-hops long. Therefore, our protocol is based on LEACH in this aspect. By using a limited and small number of hops, we are reducing the average packet delays to the sink. This is important for emergency applications that need quick reaction.

We focus on applications where events may happen at arbitrary times and where the networks need to react to those events very quickly. Such applications include chemical substance and gas detection and warning applications. Hence, we focus on reactive applications that can be served very quickly with proactive routing as in LEACH. Our protocol is designed for indoor environments in which there may be poisonous or detonating gases. We propose two protocols: R-EERP and S-EERP. In these protocols, cluster heads are selected randomly as in LEACH. In R-EERP, clustering mechanism is very similar to LEACH. In S-EERP there is a sequential cluster structure that reflects deployment of a sensor network sequentially in the rooms of the flats of a building. In S-EERP, cluster members do not change in the cluster change time. In addition, there are two threshold values, similar to the TEEN protocol in the literature, used for different goals. These are critical threshold and base threshold.

Base threshold is the minimum value desired to be sensed and reported according to the application. The values below this threshold are not taken into consideration (*i.e.*, are not reported; they are ignored at the sensor nodes). Critical threshold is a threshold value for the sensed attribute and reflects an emergency situation. Cluster heads try to send a value above the critical threshold to the sink without waiting (with as low delay as possible) in order to take the best emergency actions on the environment, in this way trying to avoid life losses.

To be energy efficient and to extend the lifetime of a sensor network, our protocol has the following features:

- The data between the critical and base thresholds are kept at the cluster heads to be sent to the base station.
- XOR operation is applied to the previous data when the data are received by cluster heads so as to decrease the number of the data packets that will be sent to the sink. Thus, duplicated data from different cluster members is sent to the sink once.

The rest of the paper is organized as follows. In Section 2 we briefly describe some related work. In Section 3 we describe our proposed routing protocols in detail. In Section 4, we provide our simulation results performed to analyze and evaluate our protocols. In Section 5, we give our conclusions.

## 2. Related Work

In recent years, quite a lot of articles have been published describing new algorithms, routing protocols and architectures aiming at WSN lifetime maximization through energy awareness. In this section, we provide a brief overview of some related research work.

The LEACH protocol [8] is the seminal protocol for both the class of hierarchical clustering protocols and proactive protocols. LEACH protocol defines the concept of round and operates in rounds. The time interval during which a new clustering is done and data gathering is performed over this new clustering several times is called a round. The LEACH protocol is designed considering that a WSN will have many rounds during its lifetime. Each round consists of two phases: cluster setup phase and steady-state phase.

Set-up Phase: While the clusters are being formed, each node decides whether or not to become a cluster head for the current round. Each node *n* chooses a random number between 0 and 1. If the chosen number is less than the threshold *T(n)*, the node becomes a cluster head for the current round. The threshold is set as Formula 1:

(1) $$T(n)=\{\frac{P}{\begin{array}{c} \mathrm{1-P}*(\mathrm{r mod 1/P})\\ 0 \end{array}}\;\;\;\;\;\;\;\;\begin{array}{c} \begin{array}{l} \mathrm{if}\;n\in G\\ \mathrm{otherwise} \end{array} \end{array}\;$$

*P* = the desired percentage of cluster-heads
*r* = the current round
*G* = the set of nodes that have not been cluster-heads in the last *1/P* rounds

Each node that has been selected as a cluster-head for the current round broadcasts an advertisement packet to the rest of the nodes. Each of the rest of the nodes selects the cluster to which it will belong based on the received signal strength of the advertisement.

Steady-state Phase: It is assumed that the nodes always have data to send during their allocated transmission time. That means the nodes are doing periodic sensing. When the data is transmitted from the nodes in a cluster to the respective cluster head. The cluster head forwards the data to the sink node after aggregation. During a round, data gathering from all sensor nodes to the sink node is performed many times. After a certain period of time, the steady-state phase ends and the network enters the set-up phase again.

The nodes that are selected as cluster-heads in a round consume more energy than the ordinary nodes. However, since the LEACH the protocol randomly selects the cluster heads at each round, cluster-head role is rotated and in this way energy consumption load is distributed among the sensor nodes in the network.

The Threshold sensitive Energy Efficient Network protocol (TEEN) was introduced in [10] and is essentially an interesting modification of the fundamental LEACH protocol (Low-Energy Adaptive Clustering Hierarchy, [8]) for reactive sensor networks [11]. The basic property of TEEN involves: (i) the clustering of nodes and (ii) the use of thresholds in order to decide whether the data should be transmitted to the sink. TEEN uses the principle of nodes’ self-organization into clusters, which is originally proposed in LEACH, in order to reduce transmissions. In addition to LEACH, at every cluster change time (*i.e.*, at every round), TEEN reports two threshold values to the nodes and the cluster heads.

Hard threshold value: This is a threshold value for the sensed attribute. In order for the node to transmit the sensed value to the cluster head, the sensed value has to be above this hard threshold value.

Soft threshold value: The sensed value is transmitted to the cluster head if there is a change compared to the previous sensed value that is greater than the soft threshold value.

The nodes continuously sense their environment. When a value greater than the threshold value is sensed, it is stored in an internal variable in the node. In the subsequent sensing activities, it is checked whether the sensed value has changed by an amount greater or equal to the soft threshold value. If so, the data is transmitted to the respective cluster head.

Our protocol also uses threshold values, but their use is different than in the TEEN protocol. We have a base threshold which is used to filter out the undesired data and in this way reduce the transmissions to the cluster heads (*i.e.*, filtering is done at the sensor nodes). We also have a *critical threshold* that is used to expedite the delivery of the data form the cluster heads to the sink node. The TEEN protocol’s thresholds are not used in the same way as they are used in our protocols. Additionally, we use filtering via a function (like XOR) in the cluster heads to reduce the amount of data sent by cluster heads, not only by the sensor nodes. Therefore, our protocols are different from the TEEN protocol.

The authors in [12] proposed a hierarchical reactive routing protocol called SHPER which achieves energy conservation by using both an energy efficient routing strategy and a power aware route selection scheme. This protocol specifies that the election of the cluster heads is not randomized. More precisely, the node elected to be the cluster head within each cluster is the one having the maximum residual energy. Furthermore, the route selection policy proposed takes into consideration both the residual energy of nodes and the energy consumption for all possible paths.

In [13], the authors proposed a protocol called EAP. In EAP, a node with a high ratio of residual energy to the average residual energy of all the neighbor nodes in its cluster range will have a large probability to become the cluster head. EAP clusters sensor nodes into groups and builds routing tree among cluster heads for energy saving communication. In addition, EAP introduces the idea of area coverage to reduce the number of working nodes within a cluster in order to prolong network lifetime.

In [14], the authors suggested an energy efficient heterogeneous clustering scheme which they call EEHC. According to this scheme, selection of cluster head is determined taking the weighted selection probability in terms of the residual energy in each node into consideration.

In [15], the authors proposed an algorithm called EECH similar to EEHC, in which there is a higher probability for a node with more energy to be selected as cluster head than a node with less energy. Furthermore, cluster heads are set to use multi-hop forwarding and routing when they would like to communicate with the base station.

Tan *et al.* [16] proposed two power efficient data gathering and aggregation protocols based on minimum spanning tree routing scheme, called PEDAP and PEDAP-PA.The basic idea is to minimize the total energy expended in a round of communication while balance the energy consumption among sensors. Simulation results show that PEDAP and PEDAP-PA perform better than LEACH and PEGASIS.

Kim *et al.* [17] suggest a protocol including some techniques such as the sleeping mode of nodes if data in the environment where detection is made is under a certain value and selecting the node with the most energy level as the cluster head in order to enhance energy efficiency.

Sajid *et al.* [18] describe Hierarchical Clustering Routing (HCR) as an extension of LEACH. In HCR, each cluster is managed by a set of associates and energy efficient clusters are retained for a longer period of time. In a variation of HCR, the base station determines the cluster formation. A genetic algorithm (GA) is used to generate energy-efficient hierarchical clusters. The base station broadcasts the GA-based clustering configuration, which is received by the sensor nodes and the network is configured accordingly.

In [19], the authors, in their algorithms based on LEACH, propose to transfer the collected data to the nearest node in terms of received signal strength by not allowing the cluster heads to directly communicate with the base station. The selected nearest node compresses the data and forwars it to base station. They state that this shortens the distance between the cluster heads and the base station and reduces energy consumption. In [20] the authors introduce a cluster head election method using fuzzy logic to overcome the drawbacks of the LEACH protocol. They investigate the use of fuzzy variables to prolong the lifetime of a homogenous network system.

Many other hierarchical routing protocols have been proposed either in the past [21–23] or more recently [24–26]. Similarly, many alternative approaches have been proposed for energy conservation in wireless sensor networks.

## 3. Efficient Energy Aware Routing Protocols: R-EERP and S-EERP

Wireless sensor networks require application specific design. For applications like detection of dangerous gases in a building or region, it is important that the network reacts to emergency cases as soon as possible, but still operates in an energy efficient way in cases where there is no emergency situation. Therefore, in this paper we propose routing protocols for such wireless sensor network applications, that can consume energy efficiently, but can also react quickly to emergency situations.

Our routing protocols are based on the LEACH protocol but have features added on top of it to be used in sensor network applications developed for dangerous gas detection. In this section, we will describe our proposed protocols in detail.

As mentioned earlier, we propose these routing protocols especially for critical applications requiring emergency response when certain events happen. For example, poisonous (like carbon monoxide) or explosive (like methane) gases existing in any indoor environment should be detected before reaching dangerous levels, and certain precautions should be taken as soon as possible. This is exactly the application scenario that we are targeting. But the protocols we propose can be used for other similar type of applications that wants quick response from time to time, even though not all the time. Hence, our solution is not limited to dangerous gas detection application.

### 3.1. Forming Clusters and Cluster Head Selection

**Figure 1.**
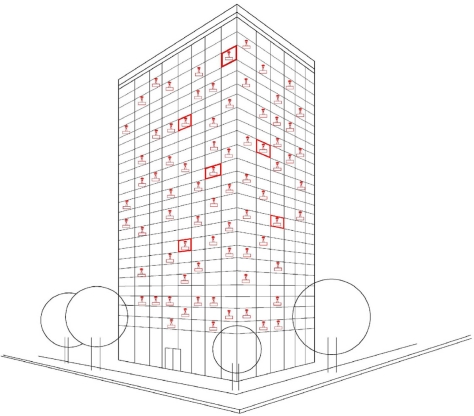
Nodes are distributed in a field within 200 × 200.

We propose two routing protocols: R-EERP and S-EERP. In our R-EERP protocol (Randomly clustered Energy Efficient Routing Protocol) the nodes are distributed randomly and uniformly in a target area. Hence the clusters are also distributed uniformly over the area. The cluster formation and cluster head selection are very similar to the ones in the LEACH protocol (Figure 1).

**Figure 2.**
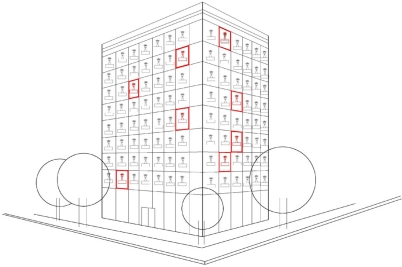
The deployment of the nodes on floors and in rooms on each of the floors.

In our S-EERP protocol (Sequentially clustered Energy Efficient Routing Protocol), the clusters are organized sequentially along the floors of a building. Hence, it has the following assumptions: (i) the environment where the sensor network is deployed is a multi-story building (a multi-floor apartment building or an office building) with a number of rooms in each floor; (ii) the nodes are deployed in each room manually; (iii) the sensor nodes in a room or in a floor constitute a cluster spanning that room or floor; and (iv) the nodes forming a cluster (which is determined at cluster setup phase) do not change while the network is operating (see Figure 2), but the node having the cluster-head role in a cluster may change at each round of reconfiguration.

### 3.2. The Use of the Critical Threshold and Base Threshold

We propose the use of two threshold values to reduce the amount of data sent to the base station, but still react to emergency cases as quickly as possible. Reacting quickly is important for wireless sensor network monitoring applications used for detection of poisonous or detonating gases which may emerge slowly or suddenly in an environment due to various reasons. The two thresholds we propose are: (1) critical threshold; (2) base threshold.

Critical Threshold: This is a critical value above which we assume that there is an emergency situation that has to be handled as quickly as possible. Hence, when the network senses a value above this threshold (*i.e.*, when a sensor node reports a value above this threshold to the cluster head), the data will be sent to the sink node without being kept waiting at the respective cluster head. It is transported towards the base station as soon as possible. What the critical threshold is depends on the application. It may vary from application to application. Therefore, here we do not specify a method about how we can set those thresholds. Critical threshold is important for the rescue teams to detect the emergency situation as soon as possible so that best emergency actions can be taken in the environment without much delay. The precautions that will be taken on time may be very important for human health and life.

Base Threshold: This is the minimum value desired to be sensed and reported to the sink node. The sensed values above the base threshold will be delivered to the base station, but there is not much hurry in delivering them. Hence the cluster head can wait such data for a while. A value below the base threshold will be ignored at the sensor nodes and will not be delivered to neither the cluster heads nor the sink node.

The nodes constantly sense their environment. But not all sensed data is delivered across the network. The sensor nodes know the base threshold (they are configured initially to know the base threshold) and filter out all sensed data below the base threshold. Only values above the base threshold are sent to the respective cluster head nodes. Therefore, a cluster head will only receive values above the base threshold. A cluster head may receive such data (values) from one or more sensor nodes in its cluster. Each cluster head collects the incoming values between the base and critical thresholds for certain amount of time. Then each cluster head compresses (or filters out with a filtering function like XOR) the different collected values and sends them to the base station. If there is an incoming data above the critical threshold, however, it is not aggregated or compressed; it is sent to the sink node immediately. Only the values above the critical threshold are treated like that (sent immediately). The use of critical and base threshold is suitable for applications requiring periodic reports and also requiring quick response to emergence cases.

The TEEN protocol [10] uses also some thresholds. But they are used in a different manner. The main drawback of TEEN is that, if the thresholds are not reached, the nodes will never communicate; the user will not get any data from the network at all and will not come to know even if all the nodes die. The base threshold in our protocol removes this deficiency of [10].

### 3.3. Avoiding Sending the Duplicated Data

In a sensor network, close-by nodes may fall into the same cluster and sense similar data. Then they may try to send their data to the base station. In our proposed solution, as the cluster-heads receive data from the nodes, an XOR operation is performed on the received data with the waiting data in order to check if they are the same. If the newly received data exists among the previously sensed data, it is ignored, because the same data has been sensed before and is being kept waiting at the cluster-head to be sent to the sink. In this way, by waiting the data and comparing the incoming data with the waiting data, redundant transmissions (transmitting the same data) is prevented. This reduces the volume of traffic transported across the resource-scarce wireless sensor network. It also means less processing at the base station. If all the received data is the same with the first waited data, none of the new data is sent to the sink. In this way, the whole network consumes less energy.

There are various methods that can be used to compare the new data with the waiting data. If the information is ON/OFF information (*i.e.*, it can be represented with a single bit), then we can apply simply an XOR operation. It will XOR a new coming bit together with the existing bit (waiting data); if the result is one, the new data will also be sent (Table 1) besides the waited data in the cluster head.

Other data filtering techniques can be used as well for different data types. XOR is good for Boolean data. For byte or integer data, binary comparison can be performed and filtering out can be decided if the difference is less than a certain value.

### 3.4. Radio Model

We use the same radio model as stated in [8] for the radio hardware energy dissipation, which is shown in Figure 3. This radio model has been widely adopted in several studies [13,14,17,18] and the simulation parameters are listed in Table 2. Transmitting (*ETx*) and receiving energy costs (*ERx*) are calculated as follows:

(2) $$\begin{array}{l} \;\;\;\;\;\;E_{\mathrm{Tx}}(k,d)=kE_{\mathrm{elec}}+k\varepsilon_{\mathrm{friss}}d^{2}\mathrm{: d}<d_{\mathrm{crossover}}\\ E_{\mathrm{Tx}}(k,d)=kE_{\mathrm{elec}}+k\varepsilon_{\mathrm{two-ray}}d^{4}\mathrm{: d}\geq d_{\mathrm{crossover}} \end{array}$$

For the receiving cost:

(3) $$E_{\mathrm{Rx}}(k)=k.E_{\mathrm{elec}}$$

where:

*E_elec_* is the transmitter circuitry dissipation per bit,
ε_amp_ is the transmit amplifier dissipation per bit per square meter.

*k* is the length of the message in bits, *d* is the distance between transmitter and receiver node, and *d_crossover_* as the distance threshold value to decide on the radio propagation model. If *d < d_crossover_*, the free space model is adopted, otherwise if *d ≥ d_crossover_*, the two-ray model is adapted (see Figure 3 and Table 2).

## 4. Simulation Analysis

In this section, we evaluate the performance of our R-EERP and S-EERP protocols via simulations. We developed a custom simulator which is written in C# program for this evaluation. We compare our results with the LEACH protocol, since LEACH is the base protocol on which we are building our protocols. Our protocols also proactive routing protocols as LEACH, but different from LEACH, our protocols support reactive applications better than LEACH and with less energy consumption. We would like to see how much we can improve LEACH with our extensions for reactive applications. We are not comparing our protocols with TEEN, since TEEN does not target the scenario that we are targeting: gas detection applications that require very fast responses from time to time and require less energy consumption. Even though TEEN reduces energy consumption by having soft/hard thresholds, it does not have a mechanism and policy for fast response in emergency situations.

### 4.1. Simulation Environment

Our simulation environment for the R-EERP, S-EERP and LEACH protocols is an area of 200 m by 200 m. All nodes are immobile. The base station is fixed and located far from the sensors. The initial energy of all of the nodes is 1.2 J. The energy consumption of a node that sends a packet is calculated with Formula (2) and the energy consumption of a node that receives a packet is calculated with Formula (3). The size of packets is assumed to be 525 bytes. Table 2 summarizes the simulation parameters [8].

For our experiments, it is considered that there could be different amounts of methane gas leakage in different areas. For this reason, it is assumed that the critical threshold value for the methane gas is 70 units and the base threshold value is 10 units. Therefore, (i) the data above 70 units will directly be transmitted to the sink without being kept waiting at cluster heads; (ii) the data below 10 units will be ignored; (iii) the data between 10 and 70 units will be sent to cluster heads when sensed; (iv) a compression/filtering operation will be performed on the data which is received at the cluster heads and in this way sending of duplicated data to the sink node will be prevented. Methane gas level is randomly determined between 5 and 87.5 in each 5 seconds during the simulation. The cluster heads are changed at each 10 events and the simulation ends after 1,000 rounds.

### 4.2. Simulation Results

We evaluate the performance of our protocols and the LEACH protocol from three different aspects. These are: network lifetime evaluation, data transmission and threshold value evaluation, and energy evaluation.

#### 4.2.1. Network Lifetime

We use two definitions for the network lifetime: FND (First Node Dies) and HNA (Half of the Nodes Alive). We consider network lifetime in terms of rounds. Network starts operating at round 1. We measure at which round the first node dies (FND) and up to which round at least half of the nodes are still alive.

Figure 3 shows that the protocols we developed have a better performance when compared to LEACH, because death of the first node in our protocols is more delayed when compared to the LEACH. This lifetime improvement results from two factors. It is firstly due to the use of critical and base threshold and secondly due to the fact that the sending of the duplicated data to the sink is prevented by performing a compression (eliminating redundancies) operation. Thus, the volume of traffic, which directly affects the energy consumed by the cluster heads and ordinary sensor nodes while sending their packets, is reduced. In this way, the lifetime of the network is increased significantly.

#### 4.2.2. Data Transmission and Threshold Value Evaluation

Next we analyze: (i) total number of transmissions; (ii) the number of data transmissions received by the base station; (iii) number of data values sensed but unsent as they are beneath the threshold value; (iv) number of data values between the base and critical threshold; and (v) number of data values that are sent immediately as they are above the critical value for the three routing protocols under consideration. For this experiment, we again simulated 100 different 200 m × 200 m network topologies. From Table 3, we can see that the total number of data transmissions received by the sink is much less in our protocols. Because a separate cluster is formed in S-EERP for every floor, the number of data transmissions in S-EERP is more than R-EERP.

**Figure 4.**
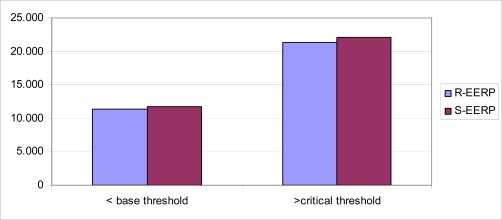
The ignored values which were below the base the base threshold and the values above the critical threshold which were immediately sent to sink.

As in Table 3, the total number of data transmissions received by the base station in our protocols is less than the LEACH protocol. Figure 4 shows that use of a threshold value is both important in terms of urgent measures and energy efficiency. The critical threshold value and above are sent to the base station without waiting them in cluster heads affects the energy efficiency in a negative way. However, network life time is significantly prolonged by ignoring the values beneath the base thresholds values and preventing duplicated values.

#### 4.2.3. Energy Evaluation

Table 4 shows the total number of dead nodes, total energy consumptions and total residual energy of the protocols after 1,000 rounds of operation. This table clearly shows that R-EERP has a much more desirable energy expenditure performance than those of LEACH and S-EERP. On average, R-EERP exhibits a reduction in energy consumption of 40 and 45 percent over S-EERP and LEACH, respectively.

## 5. Conclusions

Two energy effective routing protocols, R-EERP and S-EERP, have been presented in this paper. These protocols use critical and base threshold values. In R-EERP, nodes, clusters and cluster-heads are distributed randomly in the field and cluster heads are also selected randomly. In S-EERP, nodes, clusters and cluster heads are sequentially deployed into the floors of a building, and the cluster heads are randomly selected inside clusters whose members do not change in the floors. Use of XOR operation is proposed to filter out duplicated/redundant data received at the cluster heads.

As shown with our simulation results, the number of data transmissions sent to sink is significantly decreased with help of critical and base threshold values used by our protocols. Simulation results show that R-EERP and S-EERP had a better performance than LEACH. R-EERP achieves significant improvement in balancing energy consumption over S-EERP and LEACH.

## Figures and Tables

**Figure 3.**
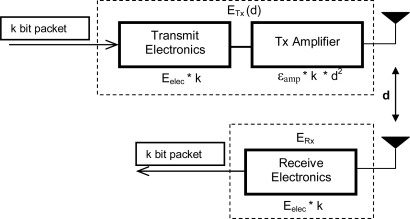
First Radio Model.

**Figure 3.**
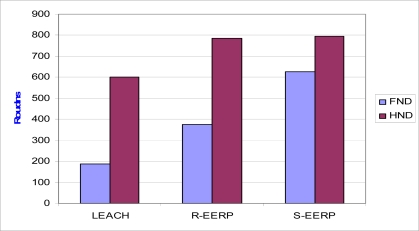
Network lifetime comparison between LEACH and our protocols.

**Table 1.** XOR Truth Table.

x	y	z
0	0	0
0	1	1
1	0	1
1	1	0

**Table 2.** Simulation parameters.

**Parameter**	**Value**
Network size	200 m × 200 m
Node count	100
Sensing range	30 m
Initial energy of each node	1.2 J
Data packet size	525 Bytes
Broadcast packet size	25 Bytes
E_elec_(Radio electronics energy)	50 nJ/bit
ε_friss_ (d < d_crossover_)	10 pJ/bit/m^2^
ε_two-ray_ (d ≥ d_crossover_)	0.0013 pJ/bit/m^4^
Threshold distance (d_crossover_)	87.01 m
Simulation rounds	1,000
P (Desired probability of cluster heads)	0.05

**Table 3.** Total number of data transmission at the whole network and received at the sink.

**Parameters**	**LEACH**	**R-EERP**	**S-EERP**
Total number of data transmission in the network	232.426	330.002	349.237
Total number of data transmission received by the sink	114.333	5.612	10.551
Number of data values sensed but unsent (<Base threshold)	-	11.310	11.759
Number of data values kept waiting at the cluster heads as they are between the base and critical thresholds	-	127.821	131.096
Number of data values sent immediately (>critical threshold)	-	21.295	22.078

**Table 4.** Number of dead nodes, total energy comsumption and residual energy after 1,000 rounds.

**Parameters**	**LEACH**	**R-EERP**	**S-EERP**
Number of Dead nodes	62	32	60
Total Energy Consumed (Joule)	109,89	99,08	108,35
Total Residual Energy (Joule)	10,11	20,92	11,65
